# Wildcat wellness coaching feasibility trial: protocol for home-based health behavior mentoring in girls

**DOI:** 10.1186/s40814-016-0066-y

**Published:** 2016-06-01

**Authors:** Brooke J. Cull, Sara K. Rosenkranz, David A. Dzewaltowski, Colby S. Teeman, Cassandra K. Knutson, Richard R. Rosenkranz

**Affiliations:** 1 Department of Food, Nutrition, Dietetics and Health, Kansas State University, 212 Justin Hall, 1324 Lovers Lane, Manhattan, KS 66506 USA; 2 Department of Kinesiology, Kansas State University, 1A Natatorium, 920 Denison Avenue, Manhattan, KS 66506 USA

**Keywords:** Children, Primary prevention, Role modeling, Social cognitive theory, Motivational interviewing

## Abstract

**Background:**

Childhood obesity is a major public health problem, with one third of America’s children classified as either overweight or obese. Obesity prevention and health promotion programs using components such as wellness coaching and home-based interventions have shown promise, but there is a lack of published research evaluating the impact of a combined home-based and wellness coaching intervention for obesity prevention and health promotion in young girls. The main objective of this study is to test the feasibility of such an intervention on metrics related to recruitment, intervention delivery, and health-related outcome assessments. The secondary outcome is to evaluate the possibility of change in health-related psychosocial, behavioral, and biomedical outcomes in our sample of participants.

**Methods/design:**

Forty girls who are overweight or obese (aged 8–13 years) will be recruited from a Midwestern college town. Participants will be recruited through posted flyers, newspaper advertisements, email, and social media. The volunteer convenience sample of girls will be randomized to one of two home-based wellness coaching interventions: a general health education condition or a healthy eating physical activity skills condition. Trained female wellness coaches will conduct weekly hour-long home visits for 12 consecutive weeks. Assessments will occur at baseline, post-intervention (3 months after baseline), and follow-up (6 months after baseline) and will include height, weight, waist circumference, body composition, pulmonary function, blood pressure, systemic inflammation, physical activity (Actical accelerometer), and self-reported survey measures (relevant to fruit and vegetable consumption, physical activity, and quality of life).

**Discussion:**

This study will evaluate the feasibility of home-based wellness coaching interventions for overweight and obese girls and secondarily assess the preliminary impact on health-related psychosocial, behavioral, and biomedical outcomes. Results will provide information regarding the feasibility of this new model for use in girls as an approach to reduce the burden of overweight and obesity toward the prevention of chronic disease.

**Trial registration:**

NCT01845480

**Electronic supplementary material:**

The online version of this article (doi:10.1186/s40814-016-0066-y) contains supplementary material, which is available to authorized users.

## Background

Approximately one out of every three children in the USA is overweight or obese [1]. Obese children are more likely than healthy weight children to have detrimental health outcomes, including high blood pressure, dyslipidemia, type 2 diabetes, and psychosocial problems [2–4]. Additionally, overweight and obese children are at a significantly greater risk of adult obesity, compared to children at a healthy weight [5]. This tracking of obesity into adulthood may lead to the development of more severe health outcomes [6]. In addition to health problems related to obesity, there is considerable financial burden associated with childhood obesity; medical costs from youth into adulthood are estimated at approximately $19,000 more per obese child, in comparison with healthy weight children [7]. As such, early interventions that target obesity treatment and prevention are warranted.

Successful childhood obesity interventions aim to regulate body fat and weight, while allowing for further growth [8] in ways that promote the persistence of healthful behaviors throughout the developmental years [9]. The goal is typically not weight loss but rather to achieve increases in height while slowing weight gain. Secondary prevention measures may focus on reducing the impact of childhood obesity by slowing or reversing the weight gain through the building of healthful behaviors. Secondary prevention techniques may include self-efficacy and skill-building for behaviors related to nutrition and physical activity [10]. Given that obesity may result from prolonged positive energy imbalance, interventions typically promote increased energy expenditure or appropriately balanced caloric intake, which can each be considered independent avenues to improve obesity outcomes. It is critical that childhood obesity interventions are handled with “kid gloves,” in that great care is taken to not stigmatize the individuals, and to ensure that quality of life is considered alongside weight status and healthful behaviors.

Both boys and girls are impacted by problems associated with obesity and could benefit from interventions and programs targeting health promotion. Girls, in particular, may do well with targeted health promotion and obesity prevention programs for a number of reasons. First, they are less physically active than boys, and this comparison becomes more disparate with increasing age, not only in terms of overall amount of physical activity but also in vigorous intensity physical activity [11]. Second, overweight and obese adolescent girls are more likely than healthy weight girls to use dysfunctional methods to try to control their weight [12]. Given the dysfunctional weight control efforts and special concerns within this population, it makes sense to develop health promotion and obesity prevention interventions that are uniquely tailored to meet the needs of girls.

Obesity prevention efforts, both primary and secondary, have been studied in a variety of settings, including during and outside of the school day. Given that children are exposed to so many different settings over the course of their childhood, there are a host of factors that may play a role in the potential of a given environment to be obesogenic. Specifically, the home environment functions as a major influence in a child’s dietary and physical activity behaviors [13], with US children consuming approximately two thirds of their daily calories in the home [14] and the physical and social environment of the home playing a role in children’s physical activity and sedentary levels [15]. Evidence shows that obesity prevention interventions delivered within the home setting can be effective in reducing body mass index in youth, specifically when education sessions and information are provided regarding healthful behavior change in physical activity and nutrition [16]. It has been suggested that obesity prevention programs may not be effective or sustainable without impacting the home environment of a child [17]. Additionally, interventions conducted within the home offer certain advantages when compared to center-based intervention delivery. Potential barriers to program participation can be limited with a home-based intervention, including location, transportation, childcare, and work obligations [18]. Additionally, it has been shown that attendance for center-based obesity prevention and treatment interventions tends to drop off quickly, with many barriers to participation being cited [19]. As a result, there is a need for additional research examining the impact of childhood health promotion and obesity interventions that are delivered within the home setting, where participation barriers can be minimized.

One type of health promotion and obesity prevention strategy that has shown success is behavior change through wellness coaching, which is a relatively new and emerging research area. In the wellness coaching model, individuals work with coaches to determine personal goals in a client-centered setting, using steps that lead to sustainable behavior change [20]. Successful wellness coaching practices have utilized several theoretical models and techniques, including social cognitive theory, self-determination theory, and motivational interviewing [21]. Components of effective wellness coaching may include client-chosen goal setting, affirmation, and activities to develop self-efficacy and intrinsic motivation. The ways in which wellness coaching has been implemented are quite diverse, as there has been success across various age groups and delivery sources (by phone, in person, online). Wellness coaching has shown promise for improving health behaviors related to chronic disease prevention [22], childhood obesity [23], dietary intake, and physical activity [24], among other health-related outcomes. Nutrition education combined with behavior change counseling has been shown to reduce body mass index in overweight and obese adolescents [25]. Additionally, a health coaching intervention that focused on nutrition and was delivered to families resulted in improved dietary intake and weight control [24]. A recent review concluded that health coaching can be effective for weight control, physical activity levels, and improved physical health, among other positive outcomes [26]. Coaching strategies may range from teaching clients general health education to more comprehensive plans that integrate skill-building and mastery experiences. Because education alone is typically not sufficient for behavior change, the skills-based component offers opportunity for capacity building, self-efficacy, self-regulation, and environmental change [27]. Although self-regulation skills can be developed within various childhood settings, wellness coaching that helps build these skills within the home environment provides a unique opportunity to target distinct mediators of behavior change that are most relevant to the home. Specifically, when children are able to learn self-regulation and environmental change skills that they can apply within the home setting, they may be better equipped to influence, control, and improve their home environment and associated health-related behaviors.

Although there is evidence to suggest that wellness coaching and home-based interventions can be effective as separate strategies for health promotion and obesity prevention, there is a lack of research examining the combination of the two, specifically a one-on-one, home-based, wellness coaching intervention for young girls. Therefore, our objective is to evaluate a new model, which consists of a wellness coaching intervention delivered within the home environment for young females. For the purposes of this paper, the term home-based refers to interventions delivered within the home and should not be confused with interventions delivered in alternate settings that intend to impact the home as a behavior setting.

The primary objective of this one-on-one, home-based wellness coaching intervention is to evaluate the feasibility metrics related to recruitment, intervention delivery, and health-related outcome assessments. These primary outcome feasibility assessments will include:

- The number of participants we are able to recruit from the local area and surrounding towns
- The length of time required to recruit 40 eligible participants
- The number and percentage of wellness coaching sessions delivered within the home setting
- Fidelity of intervention delivery
- Participant and parent satisfaction with intervention delivery
- Adverse effects associated with the intervention
- The number of participants who complete post-intervention and follow-up laboratory assessments

As a secondary outcome, we also seek to evaluate the preliminary impact of the intervention on change in girls’ health-related psychosocial, behavioral, and biomedical outcomes. Two arms of the intervention will be implemented: a healthful eating and physical activity (HEPA) skills condition and an active comparison group representing a general health education (HE) condition. We hypothesize that both intervention arms will be successful in recruitment and retention of participating families and delivery of the intervention sessions by trained college-aged research assistants and that both of the coaching conditions will be well received and appreciated by participating families.

## Methods/design

### Study design

**Fig. 1:**
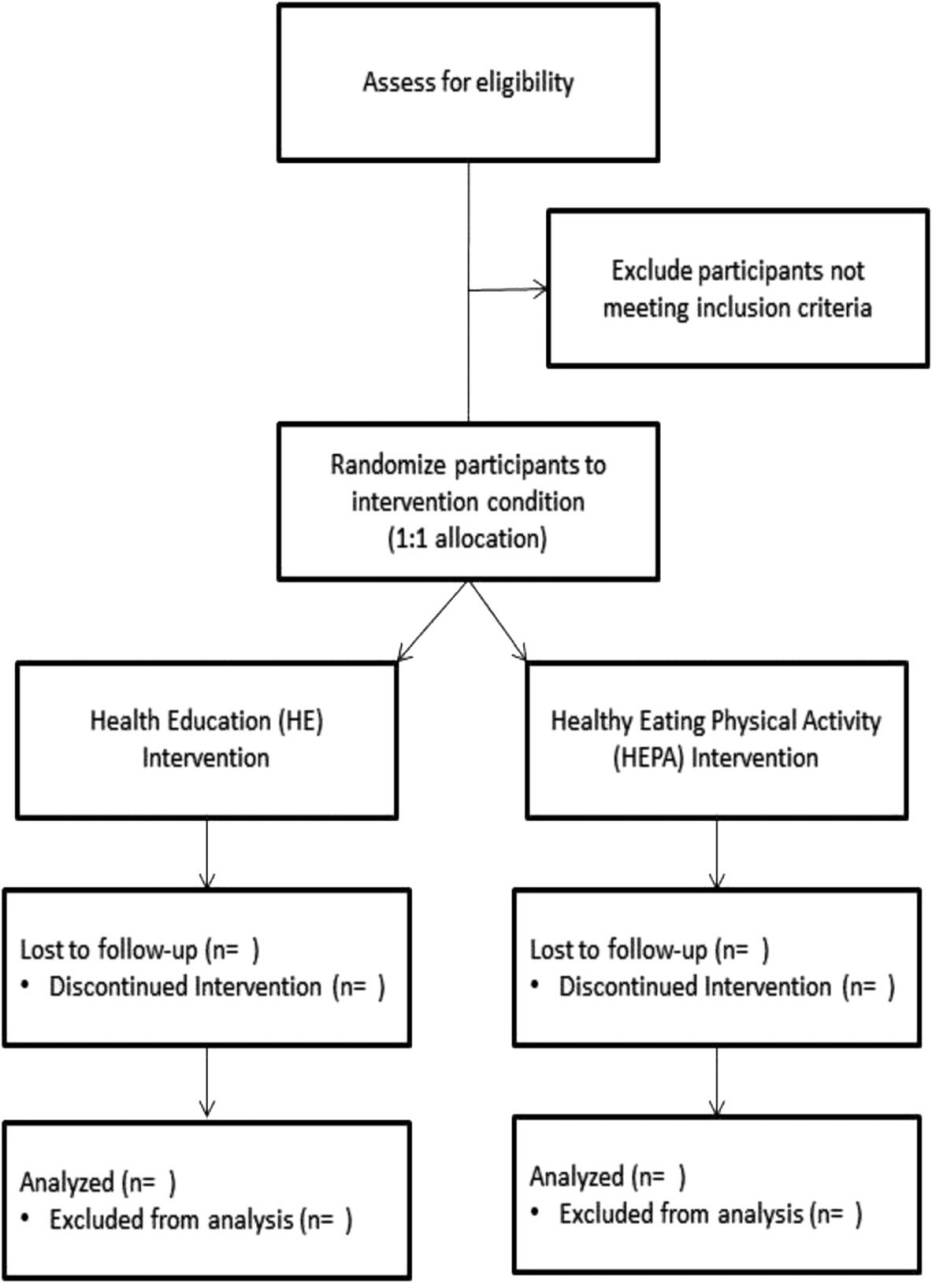
Study flow diagram for The Wildcat Wellness Coaching Trial

As shown in Fig. 1, this study is a two-arm, parallel randomized trial comparing two home-based coaching interventions on primary feasibility outcomes related to recruitment, intervention delivery and acceptance, and health outcome assessments, with secondary, preliminary measures related to health-related psychosocial, behavioral, and biomedical outcomes of female children. The home-based, wellness coaching intervention period will last for 12 weeks, with laboratory assessments at baseline, post-intervention (3 months after baseline), and at follow-up (6 months after baseline). A 6-month follow-up period was chosen to evaluate the feasibility of having participants return to the laboratory for an assessment following the conclusion of the intervention. A fully powered study examining the impact on health outcomes may employ a longer-term follow-up to assess maintenance of behavior change and subsequent changes in health outcomes.

### Study population

Participants for this study are 40 females aged 8 to 13 years. All girls will have a body mass index (BMI) at or above the 85th percentile (overweight or obese) of gender-specific, age-adjusted growth charts and must reside within 20 miles of Manhattan, KS (Kansas State University). Exclusion criteria require that the girls do not have developmental delays, psychiatric problems, or any illness, injury, or condition that prevents them from participating in moderate-to-vigorous physical activity. Finally, the participants cannot be taking weight-altering medication or be participating in any other weight control program. There will be no inclusion/exclusion criteria related to socioeconomic status or ethnicity. Parental consent and child assent will be obtained from all participants prior to their participation in the study. Kansas State University Institutional Board of Human Ethics approval has been obtained, and all study methods will comply with the Declaration of Helsinki.

### Recruitment

Parents of girls interested in a health promotion program will be recruited in various ways, including posted flyers, newspaper advertisements, social media, and word of mouth. Parents who are interested in having a daughter participate in the program will contact the principal investigator to obtain further information regarding the program and associated research study and an anticipated timeline for when their assessments and home visits will begin.

### Randomization

Rolling recruitment will be implemented, and participants meeting the inclusion criteria will be block-randomized using an online randomization program into one of two wellness coaching conditions, in blocks of four, with a 1:1 allocation ratio. The principal investigator will oversee the randomization process and will keep allocation concealed until baseline assessments have been completed. Research assistants collecting assessment data will be blinded to group randomization over the course of the study. Blinding of the study participants to their group assignment will not be possible due to the nature of the intervention itself, but participants will not know their assigned condition until after baseline assessments.

### Wellness coaching interventions

For both the HEPA skills and HE coaching intervention conditions, college-aged female research assistants will undergo ethics training, plus three 1-h coach training sessions, to serve as wellness coaches and deliver 12 weekly one-on-one intervention sessions in the home of each participating child. Materials and lessons for the wellness coaching curriculum are adapted from “Health in the Classroom” [28] and other supporting educational materials as appropriate to the topic and age group of the client. The theoretical basis for the intervention development comes from social cognitive theory [29] and the self-determination theory [30]. Social cognitive theory posits that behavior, personal factors, and the environment have reciprocal effects on one another to determine human behavior. Within this intervention, personal factors targeted are self-efficacy, observational learning, and monitoring and regulation of behavior. Constructs from the self-determination theory include autonomy, relatedness, and competence, which are also targeted within both arms of the intervention. Training will take place before coaching sessions 1, 5, and 9. The wellness coach and parent will mutually agree upon meeting times, so that at least one parent is always present in the home during the session, although the parents will not be involved in the coaching sessions. There will be flexibility in scheduling, and parents will be given the option for these times to remain the same over the course of the 12 weeks or to change as schedules require.

For the HEPA skills coaching intervention, the coaches will use motivational interviewing techniques in applying the theoretical components of role modeling, outcome expectations, social support, autonomy support, relatedness, competence, and vicarious learning to build self-efficacy and motivation and enhance behavior change to promote health and wellness. The content and activities of the 12 visits are shown in the Additional file 1: Table S1. A main theme of the home visits will be physical activity promotion and fruit and vegetable snack consumption through skill-building activities in the home environment. The wellness coaches will help the girls to set wellness goals and to self-monitor their healthful eating and physical activity behavior. The girls will be taught kitchen skills for fruit and vegetable preparation, as well as enjoyable physical activities they can do at home. Finally, the wellness coach will complete the activities alongside the girls in order to provide a source of social support and role modeling for the promotion of physical activity and healthful eating.

The HE coaching intervention, the active comparison group, is designed to help girls set goals and self-monitor their behaviors with a more general focus on overall health promotion, not specifically on physical activity and dietary intake behaviors. Wellness coaches will work to educate girls on a range of relevant health promotion behaviors including tooth brushing, smoking prevention, and physical activity. Similar to the HEPA skills intervention, coaches are meant to provide social support and role modeling for practicing healthful behaviors but will not lead physical activities or snack preparations as they do in the HEPA skills condition. Over the 12 weeks of the intervention, the HE coaching sessions will focus on the discussion and understanding of a range of health topics and health behaviors, as well as their benefits, barriers, and change strategies.

In both arms of the intervention, the coaching visits will have specifically assigned primary activities and secondary activities, along with other supplemental activities that coaches may include during their time with the girls. In addition to the scheduled activities, coaches will also integrate discussion of school, well-being, emotions, and everyday difficulties of the girls within both arms of the intervention. In the HEPA skills intervention group, discretionary time that follows completion of the lesson for the day will be spent making choices for physical activity and fruit and vegetable snack for their next session or helping with homework, playing games, doing puzzles, or talking. In the HE group, wellness coaches will be able to spend any extra time helping with homework, playing games, doing puzzles, or talking.

### Primary outcome evaluation

#### Recruitment feasibility

Recruitment feasibility will be assessed through the reach or number of participants we are able to recruit from the local area and surrounding town into the trial. Additionally, we will determine the length of time required to recruit 40 eligible participants.

#### Intervention delivery feasibility and acceptance

Intervention delivery feasibility will be assessed based on several metrics. We will determine the percentage of wellness coaching sessions delivered and completed within the home setting of each participant via coach self-report, following each session. The fidelity of the intervention delivery components will be determined based on whether or not primary and secondary activities were performed during the home-based sessions. Any adverse effects related to intervention delivery will be collected via coach report. Intervention acceptance will be measured through parent and participant satisfaction questionnaires, which will be completed during a post-intervention interview. These metrics for feasibility will allow us to assess whether or not the wellness coaching intervention can be delivered according to a weekly schedule and whether the intervention is accepted by participants and parents.

#### Health-related outcome assessment feasibility

The feasibility for assessing health outcomes will be determined by calculating the number and percentage of participants who complete the post-intervention and follow-up health assessments. These metrics will allow us to understand whether laboratory assessments can be completed during the scheduled timeframe and whether it is feasible for this sample to return to the laboratory following a no-intervention follow-up period.

### Secondary outcome evaluation

The health-related psychosocial outcomes include changes in child quality of life (measured through the PedsQL instrument), as well as self-efficacy and enjoyment of physical activity and fruits and vegetables. Health-related behavioral outcomes for this study are the change in daily physical activity and sedentary time (minutes per day, as measured by accelerometer), steps per day, and fruit and vegetable intake. Health-related biomedical outcomes include change in BMI *Z*-score from baseline, as well as changes in body fat percentage, waist circumference, blood pressure, pulmonary function, and systemic inflammation.

#### Laboratory assessment procedure

Participants and a parent will attend laboratory assessments at baseline, post-intervention (3 months after baseline), and follow-up (6 months after baseline), which consist of a battery of psychosocial, behavioral, and biomedical outcomes. The hour-long assessments will be conducted in the Physical Activity and Nutrition Clinical Research Consortium Laboratory at Kansas State University by a team of two trained graduate research assistants. The assessments will include (1) demographic and parent dietary/physical activity information, (2) quality of life questionnaire, (3) self-efficacy and enjoyment of physical activity/fruit and vegetable questionnaire, (4) child dietary intake questionnaire, (5) physical activity levels via accelerometry over a 5-day period, (6) anthropometric assessments, (7) blood pressure, (8) pulmonary function measurements, and (9) markers of systemic inflammation. The details and specific methodology for each component of the assessment follow. Following the baseline assessment period, the 12 weeks of home-based wellness visits will begin.

#### Laboratory measures

##### Demographic and parent information

Previously validated questionnaires will be used to assess parental demographics, physical activity levels [31], and fruit and vegetable consumption [32].

##### Psychological outcomes: child quality of life, self-efficacy, and enjoyment

In order to assess quality of life, the PedsQL instrument for ages 8 through 12 and the PedsQL parent-proxy report for ages 8 through 12 will be used. The PedsQL has been shown to be reliable and valid in the core quality of life areas (physical, emotional, social, school) for a pediatric population, and it is appropriate for use in clinical trials [33]. Additional self-report instruments will be used to assess participant (child) self-efficacy [34] and enjoyment [35] for physical activity and will be modified additionally to assess these constructs for fruit and vegetable consumption. Should questions or difficulties arise when girls are completing the questionnaires, research staff will provide clarification and assistance as needed.

##### Behavioral outcomes: child typical dietary intake

Together with their parent or caregiver, the girls will complete the Childhood Dietary Questionnaire [36]. The participants will be asked to report the number of times that they have consumed particular foods in the past 24 h or 7 days, depending upon the food category, in accordance with standard instructions and the format of the dietary questionnaire. The Childhood Dietary Questionnaire will be used to assess dietary intake and is a validated tool to measure intake in the categories of fruits and vegetables, dairy fat, sweetened beverages, and non-core foods. This questionnaire is not meant to assess caloric intake but rather intake in specific categories of diet over a specified time period. From this questionnaire, we will be able to assess fruit and vegetable consumption by determining the number of types and various categories of these foods that the participants consumed.

##### Behavioral outcomes: physical activity

Actical physical activity monitors (Respironics Inc., Bend, OR, USA) will be used to measure the physical activity behaviors of the participants. These are small pedometer-like devices that can be worn on the wrist. Participants will wear an Actical accelerometer on their non-dominant wrist for seven continuous days at each assessment period. The research assistant will apply a locking nylon band to secure the device to each girl’s wrist, ensuring continual wear. The accelerometers will be initialized to record data in 15-s epochs. After 1 week of wear, participants will return to the laboratory to have their wrist band removed, and the data will be downloaded and analyzed. Upon data download and analysis, device location will be specified as wrist, and the default adolescent Actical software cut points of 0.01 kcal/min/kg (sedentary/light), 0.04 kcal/min/kg (light/moderate), and 0.10 kcal/min/kg (moderate/vigorous) will be used. After the participant has worn the device, the data can be downloaded for an analysis of daily information regarding total steps, total time, and percentage of time spent engaged in sedentary, light, moderate, and vigorous physical activities.

##### Biomedical outcomes: anthropometrics

For all body composition assessments, participants will be asked to remove their shoes and any outer clothing or heavy garments. The research assistant taking measurements will be the same for all assessment periods and for all participants. Height will be measured to the nearest 0.1 cm with a portable stadiometer (Invicta Plastics, Leicester, England), and weight will be measured to the nearest 0.1 kg with a digital scale (Pelstar LLC, Alsip, IL, USA). BMI will be calculated as weight (kg) divided by height (m) squared and then converted to age-sex percentiles via CDC growth charts [37]. Waist circumference will be measured via a non-elastic Gulick tape measure in the horizontal plane at the iliac crest following a normal exhalation. All anthropometric measurements will be taken in duplicate, and a third measurement will be recorded if the values differ by more than 0.5 cm or 0.5 kg. The two values that are within the acceptable difference range will then be averaged and subsequently used in analyses.

A dual-energy x-ray absorptiometry (DEXA) scan will be performed to assess body composition, and relevant information will be collected, including fat, lean, and bone tissue content (GE Lunar Prodigy, Madison, WI, USA). Participants will be asked to remove heavy outer clothing, everything from their pockets, and any metal jewelry before they lie down on top of the x-ray bed. During the approximately 5-min scan, participants will be instructed to lie as still as possible. Additionally, bioelectrical impedance analysis (RJL Systems, Quantum II, Clinton Twp, MI, USA) will be performed to determine the amount of body water and resulting body composition of participants. The top of the right hand and foot will be cleaned with an alcohol swab, and the area will be given time to dry. An electrode tab will be placed in one of four specified locations on the same side of the body: around the proximal portion of the middle finger, across the top of the wrist, across the top of the ankle, and on the joint between the metatarsal phalanges on the top of the foot. The BIA wires will be connected to the electrode tabs, and readings will be recorded for both resistance and reactance. These values will be entered into the prediction equation using RJL software to calculate body composition.

##### Biomedical outcomes: blood pressure

Blood pressure will be measured with an automated blood pressure device (Omron Healthcare, model HEM-907XL, Vernon Hills, IL, USA) to obtain measurements for systolic and diastolic blood pressure, as well as pulse rate. A research assistant will measure blood pressure in a quiet room while the participant is in a seated position with both feet resting on the floor. The appropriate-sized blood pressure cuff will be applied to the upper left arm, so that the bottom of the cuff is approximately 1 in. above the elbow crease. Participants will be instructed to remain relaxed and still during the measurement. There will be at least 1 min between blood pressure measurements. Blood pressure assessments will be taken in duplicate, and a third measurement will be taken if the values for systolic or diastolic recordings differ by more than 5 mmHg.

##### Biomedical outcomes: pulmonary function

Pulmonary function will be assessed via a handheld spirometer and disposable mouthpiece (MIR winspiroPRO, version 4.4.1, Waukesha, WI). Pulmonary function outcomes of interest will include peak values of forced vital capacity (FVC) (L), forced expiratory volume over FVC (FEV1/FVC), and forced expiratory flow volume in 1 s (FEV_1_, L/s), and values will be compared to norms for age, height, ethnicity, and sex. For the measurement of lung capacity and functionality, participants will hold the spirometer while in a seated position with feet flat on the floor with their back supported and straight. The participants will wear a noseclip to ensure that all expired air is collected through the mouthpiece. PFTs will be performed, according to the American Thoracic Society criteria [38], twice, and a third measurement will be taken if FVC differs by more than 150 mL between measurements.

##### Biomedical outcomes: systemic inflammation

A marker of systemic inflammation, C-reactive protein, will be assessed via a passive drool sample. Prior to collection of the passive drool sample, participants will be asked to rinse their mouth thoroughly with water. Saliva will be collected using the Saliva Collection Aid (Salimetrics, item no. 5016.02) into a polypropylene vial (Salimetrics, item no. 5002.02). Participants will be instructed to provide approximately 1.0 mL in a 2.0-mL cryovial through the passive drool process. We will store the collected samples in a sub-60 °C freezer for storage until processing and analysis takes place. The thawed samples will be processed using enzyme-linked immune sorbent assay (ELISA) (MyBioSourceMBS163178). In our previous work, intra-assay precision was coefficient of variation (CV) < 10 % and inter-assay precision was CV < 12 %. Sensitivity of the assay was 0.01 mg/L. On the day of the assay, the samples will be thawed, vortexed, and centrifuged at 1500×*g* (at 3000 rpm) for 15 min to remove mucins and other particulate matter.

### Cost-effectiveness evaluation

Over the course of the intervention, we will calculate monetary and time costs required for delivering the intervention components and performing laboratory assessments. This will inform future estimates of cost-effectiveness for a larger-scale intervention.

### Statistical analyses

Feasibility outcomes (retention, participation, compliance, etc.) will be assessed through descriptive statistics. Data will be analyzed using SPSS statistical software (version 22.0). Despite a lack of power to detect differences between the two intervention arms, we will perform a preliminary assessment of change in psychosocial, behavioral, and biomedical outcomes. There will be an emphasis on presenting the descriptive statistics for health-related outcomes, with summary statistics and confidence intervals reported. An alpha of 0.05 will be used for all analyses, and effect sizes for future sample size calculations will be calculated on potential primary outcome measures by subtracting baseline means from post-intervention and follow-up means, divided by the appropriate pooled standard deviation. Where appropriate, results will be presented as point estimates, supported by 95 % confidence intervals.

## Discussion

With the results from this study, we will determine whether or not a home-based wellness coaching program is feasible for use with female children. A secondary outcome will be to evaluate the possibility of change in health-related psychosocial, behavioral, and biomedical outcomes through each intervention condition. Since this is a feasibility trial, we want to assess outcomes for both of the treatment arms of the intervention. The intervention conditions will be evaluated for future potential, so that they may be integrated most effectively into a future fully powered randomized controlled trial. For this type of intervention, we will recruit participants through the parents, who are concerned, at least to some extent, with the health of their children. As such, it may not be an ethical or viable option to offer no type of intervention. Because of this, our active comparison group represents general health behavior mentoring but does not focus specifically on physical activity and nutrition. This condition of health behavior mentoring used in our active comparison group, while not precisely current practice, represents more of a “standard” than the hands-on approach used in the HEPA intervention arm. Since this is a new combination of a one-on-one wellness coaching intervention delivered within the home setting to girls, there is no current literature available regarding effect sizes needed for future power calculations. As such, we will assess the preliminary impact on health-related outcomes to more appropriately inform a future fully powered trial. Using a 6-month follow-up assessment will allow us to evaluate the feasibility of having participants return to the laboratory following a no-contact period, as well as the potential for lasting impacts resulting from the study.

An additional consideration when developing an intervention with multiple outcome measures is participant burden. The inclusion of the health outcome measures has been a very successful recruitment tool in the past, since parents will be provided with a results packet at the conclusion of the intervention. We are able to provide health information for their children at no cost. In our experience, participants have viewed the battery of health assessments (lasting around 1 h) as a benefit, rather than a burden. Additionally, participants are also receiving a free wellness coach in their home for 12 weeks. For the families who have completed the intervention, we have qualitative data from parents and girls expressing that the overall process has been a positive experience.

The proposed home-based wellness coaching model, consisting of both the health education and healthy eating and physical activity skills conditions, may serve as a novel approach to overcome difficulties of reaching this specific population for early health promotion and obesity prevention intervention. Such early intervention is needed, due to the high prevalence of overweight and obesity and the associated costs, both physical and financial. Additionally, the National Prevention Strategy is codified within the Affordable Care Act and seeks to integrate effective prevention strategies to promote improved health and well-being [39]. As such, there is now a much better platform for prevention within the USA, and obesity prevention through home-based wellness coaching may align well with those national efforts. Creating sustainable behavior change through wellness coaching may play a key role in reducing the burden of childhood obesity. If feasible, the model will be further evaluated in a fully powered randomized controlled trial study design.

## Additional file

Additional file 1: Table S1. Weekly coaching schedule. Breakdown of weekly coaching schedule and activities by condition.

## References

[1] Ogden CL, Carroll MD, Kit BK, Flegal KM (2014). Prevalence of childhood and adult obesity in the United States, 2011-2012. JAMA.

[2] Ebbeling CB, Pawlak DB, Ludwig DS (2002). Childhood obesity: public-health crisis, common sense cure. Lancet.

[3] Freedman DS, Dietz WH, Srinivasan SR, Berenson GS (1999). The relation of overweight to cardiovascular risk factors among children and adolescents: the Bogalusa Heart Study. Pediatrics.

[4] Schwimmer JB, Burwinkle TM, Varni JW (2003). Health-related quality of life of severely obese children and adolescents. JAMA.

[5] Singh AS, Mulder C, Twisk JW, Van Mechelen W, Chinapaw MJ (2008). Tracking of childhood overweight into adulthood: a systematic review of the literature. Obes Rev.

[6] Srinivasan SR, Bao W, Wattigney WA, Berenson GS (1996). Adolescent overweight is associated with adult overweight and related multiple cardiovascular risk factors: the Bogalusa Heart Study. Metabolis.

[7] Finkelstein EA, Graham WCK, Malhotra R (2014). Lifetime direct medical costs of childhood obesity. Pediatrics.

[8] Rees JM (1990). Management of obesity in adolescence. Med Clin N Am.

[9] Epstein LH, Myers MD, Raynor HA, Saelens BE (1998). Treatment of pediatric obesity. Pediatrics.

[10] Hoelscher DM, Butte NF, Barlow S, Vandewater EA, Sharma SV, Huang T (2015). Incorporating primary and secondary prevention approaches to address childhood obesity prevention and treatment in a low-income, ethnically diverse population: study design and demographic data from the Texas Childhood Obesity Research Demonstration (TX CORD) study. Child Obes.

[11] Trost SG, Pate RR, Sallis JF, Freedson PS, Taylor WC, Dowda M, Sirard J (2002). Age and gender differences in objectively measured physical activity in youth. Med Sci Sports Exerc.

[12] Neumark-Sztainer D, Story M, Hannan PJ, Perry CL, Irving LM (2002). Weight-related concerns and behaviors among overweight and nonoverweight adolescents. JAMA Pediatr.

[13] Spurrier NJ, Magarey AA, Golley R, Curnow F, Sawyer MG (2008). Relationships between the home environment and physical activity and dietary patterns of preschool children: a cross-sectional study. Int J Behav Nutr Phys Act.

[14] Adair LS, Popkin BM (2005). Are child eating patterns being transformed globally?. Obes Res.

[15] Tandon P, Grow HM, Couch S, Glanz K, Sallis JF, Frank LD, Saelens BE (2014). Physical and social home environment in relation to children’s overall and home-based physical activity and sedentary time. Prev Med.

[16] Stark LJ, Spear S, Boles R, Kuhl E, Ratcliff M, Scharf C (2011). A pilot randomized controlled trial of a clinic and home-based behavioral intervention to decrease obesity in preschoolers. Obesity.

[17] Rosenkranz RR, Dzewaltowski DA (2008). Model of the home food environment pertaining to childhood obesity. Nutr Rev.

[18] Conwell LS, Trost SG, Spence L, Brown WJ, Batch JA (2010). The feasibility of a home-based moderate-intensity physical activity intervention in obese children and adolescents. Br J Sports Med.

[19] Wolcott D, Huberty J, McIlvain H, Rosenkranz R, Stacy R (2011). Changing health behaviors: exploring families’ participation in a family-based community intervention for overweight/obese children. Childhood Obesity (Formerly Obesity and Weight Management).

[20] Wolever RQ, Eisenberg DM (2011). What is health coaching anyway? Standards needed to enable rigorous research: comment on “evaluation of a behavior support intervention for patients with poorly controlled diabetes”. Arch Intern Med.

[21] National Consortium for Credentialing of Health & Wellness Coaches. http://www.ncchwc.org/ (2015). Accessed 22 Feb 2015. 10.7453/gahmj.2014.062 PMC4311565 25694854

[22] Lawn S, Shoo A (2010). Supporting self-management of chronic health conditions: common approaches. Patient Educ Couns.

[23] Nguyen B, Shrewsbury VA, O’Connor J, Steinbeck KS, Lee A, Hill AJ (2012). Twelve-month outcomes of the loozit randomized controlled trial: a community-based healthy lifestyle program for overweight and obese adolescents. Arch Pediatr Adolesc Med.

[24] Paineau DL, Beaufils F, Boulier A, Cassuto DA, Chwalow J, Combris P (2008). Family dietary coaching to improve nutritional intakes and body weight control: a randomized controlled trial. Arch Pediatr Adolesc Med.

[25] Rice J, Thombs D, Leach R, Rehm R. Successes and barriers for a youth weight-management program. Clinical Pediatrics. 2007;47:143–47. 10.1177/0009922807306168 17766579

[26] Kivelä K, Elo S, Kyngäs H, Kääriäinen M (2014). The effects of health coaching on adult patients with chronic diseases: a systematic review. Patient Edu Couns.

[27] Contento IR. Nutrition education: linking research, theory, and practice. Burlington, MA: Jones and Bartlett Learning; 2007. 18296331

[28] Health in the Classroom. https://www.healthiergeneration.org/take_action/schools/health_education/health_in_the_classroom/. Accessed 20 July 2015.

[29] Bandura A (1998). Health promotion from the perspective of social cognitive theory. Psychol Health.

[30] Deci EL, Ryan RM (2008). Self-determination theory: a macrotheory of human motivation, development, and health. Canadian Psychology/Psychologie canadienne.

[31] Prochaska JJ, Sallis JF, Long B (2001). A physical activity screening measure for use with adolescents in primary care. Arch Pediatr Adolesc Med.

[32] Prochaska JJ, Sallis JF (2004). Reliability and validity of a fruit and vegetable screening measure for adolescents. J Adolesc Health.

[33] Varni JW, Seid M, Kurtin PS (2001). PedsQL™ 4.0: reliability and validity of the pediatric quality of life inventory™ version 4.0 generic core scales in healthy and patient populations. Med Care.

[34] Dzewaltowski DA, Geller KS, Rosenkranz RR, Karteroliotis K (2010). Children’s self-efficacy and proxy efficacy for after-school physical activity. Psychol Sport Exerc.

[35] Dishman RK, Hales DP, Sallis JF, Saunders R, Dunn AL, Bedimo-Rung AL, Ring KB (2009). Validity of social-cognitive measures for physical activity in middle-school girls. J Pediatr Psychol.

[36] Magarey A, Golley R, Spurrier N, Goodwin E, Ong F (2009). Reliability and validity of the children’s dietary questionnaire; a new tool to measure children’s dietary patterns. Int J Pediatr Obes.

[37] Kuczmarski RJ, Ogden CL, Grummer-Strawn LM, Flegal KM, Guo SS, Wei R (2000). CDC growth charts: United States. Adv Data.

[38] Miller MR, Hankinson J, Brusasco V, Burgos F, Casaburi A, Coates R (2005). Standardisation of spirometry. Eur Respir J.

[39] National Prevention Council (2011). National Prevention Strategy.

