# Supplementary material for: Wildcat wellness coaching feasibility trial: protocol for home-based health behavior mentoring in girls
Source: Pilot Feasibility Stud. 2016 Jun 1;2:26. doi: 10.1186/s40814-016-0066-y (PMC5154020; doi:10.1186/s40814-016-0066-y)
Supplement: Additional file 1: Table S1. — Weekly coaching schedule. Breakdown of weekly coaching schedule and activities by condition. [file 40814_2016_66_MOESM1_ESM.pdf]

Table S1 Weekly coaching schedule

	Health Education (HE)		Healthful Eating & Physical Activity Skills (HEPA)	
	Primary Activities	*Secondary Activities*	*Primary Activities*	*Secondary Activities*
**Week 1**	-Ice Breaker<br>-SMART goal setting for school or health	-Create a picture representing goal	-Ice breaker<br>-Walk & talk (client ≥2000 steps)<br>-Prepare "ants on a log recipe" (or another F&V, using good food safety practices)	-SMART goal setting
**Week 2**	-Discussion and demonstration of good table manners, etiquette, place setting	-Family meal role-playing with or without a snack	-Choice of fun physical activity (client ≥2000 steps)	- Prepare chosen fruit & vegetable snack recipe (>50% fruits &/or vegetables, ≥taste, 200-250kcals)
**Week 3**	-Discussion of breakfast;<br>-Demonstration of basic food safety	-Draw or create representation of a healthy breakfast	-Choice of fun physical activity (client ≥2000 steps)	- Prepare chosen fruit & vegetable snack recipe (>50% fruits &/or vegetables, >taste, 200-250kcals)
**Week 4**	-Discussion of sweets, sugary drinks, dental health<br>-Demonstration of brushing, flossing, swish & swallow	-Mouth and teeth quiz	-Choice of fun physical activity (client ≥2000 steps)	- Prepare chosen fruit & vegetable snack recipe (>50% fruits &/or vegetables, >taste, 200-250kcals)
**Week 5**	-Discussion of physical fitness; inventory home opportunities for physical activity	-Revisit SMART goal(s) from week 1, draw motivational picture or poster to reinforce goal(s)	-Choice of fun physical activity (client ≥2000 steps)	-Revisit SMART goal(s) from week 1; Prepare chosen fruit & vegetable snack recipe<br>(>50% fruits &/or vegetables, >taste, 200-250kcals)
**Week 6**	-Discussion of bullying; tell or show stories of	-Role play both sides of bullying, maybe use	-Choice of fun physical activity	- Prepare chosen fruit & vegetable snack recipe

	how other kids deal with bullies	action figures, dolls, or play-dough actors, etc.	(client ≥2000 steps)	(>50% fruits &/or vegetables, >taste, 200-250kcals)
**Week 7**	-Discussion of healthy snacking; demonstration of portion sizes	-Take inventory of foods available and accessible at home, categorize according to traffic-light: go-slow-whoa	-Choice of fun physical activity (client ≥2000 steps)	- Prepare chosen fruit & vegetable snack recipe (>50% fruits &/or vegetables, >taste, 200-250kcals)
**Week 8**	-Discussion of smoking; tell or show stories of how other kids deal with peers to remain smoke-free	-Role play peer pressure for smoking, use action figures, dolls, or play-dough actors	-Choice of fun physical activity (client ≥2000 steps)	- Prepare chosen fruit & vegetable snack recipe (>50% fruits &/or vegetables, >taste, 200-250kcals)
**Week 9**	-Discussion of internet/online dos and don’ts, online bully prevention and safety; tell or show stories of kids who were mistreated through internet	-Revisit SMART goal(s) from week 1, draw motivational picture or poster to reinforce goal(s)<br>- Play online or surf internet sites to identify advertising, data mining, etc.	-Choice of fun physical activity (client ≥2000 steps)	-Revisit SMART goal(s) from week 1; Prepare chosen fruit & vegetable snack recipe<br>(>50% fruits &/or vegetables, >taste, 200-250kcals)
**Week 10**	- Discussion of self-esteem and body image; role play ways to deal with others who make us feel bad about our selves	- Mirror mirror project, or make a list with client of all the things she is good at doing, what she likes about herself, friends and family who believe in her, and a separate list of what she wants to work on to feel better about herself.	-Choice of fun physical activity (client ≥2000 steps)	- Prepare chosen fruit & vegetable snack recipe (>50% fruits &/or vegetables, >taste, 200-250kcals)
**Week 11**	- Discussion of eating disorders, including anorexia,	- Use glamour magazines to look at how women are	-Choice of fun physical activity (client ≥2000 steps)	- Prepare chosen fruit & vegetable snack recipe

	bulimia, compulsive overeating (keep focus on healthful eating and physical activity); role play ways to express concern for a friend who is showing signs of dysfunctional eating or activity	pictured and discuss how images are manipulated (optional- watch Dove video)		(>50% fruits &/or vegetables, >taste, 200-250kcals)
**Week 12**	-Discussion of all previous health topics; role reversal of client teaching what they have learned over past weeks for each topic	-Revisit SMART goal(s) from previous weeks, set new goal for next 12 weeks, draw motivational picture or poster to reinforce goal(s)<br>-Praise successes and trouble-shoot failures<br>-Talk about how to maintain what has been learned	-Discussion of all previous physical activities and snack recipes; Client can talk about likes, dislikes, which ones they'll try to keep doing in the future	-Revisit SMART goal(s) from previous weeks, set new goal for next 12 weeks; Prepare chosen fruit & vegetable snack recipe (>50% fruits &/or vegetables, >taste, 200-250kcals)<br>-Choice of fun physical activity (client $\geq$2000 steps)
